# Impact of Diets Supplemented With Low Glycemic Index Starch on Physiology and Locomotor Abilities of Mice

**DOI:** 10.1155/jt/9947667

**Published:** 2025-12-12

**Authors:** Khanh Son Trinh, Minh Hai Nguyen, Vinh Tien Nguyen

**Affiliations:** ^1^ Faculty of Chemical and Food Technology, Ho Chi Minh City University of Technology and Education, Ho Chi Minh City, 70000, Vietnam, hcmute.edu.vn

## Abstract

This study explores the effects of a low glycemic index starch (HR0) produced through double thermal retrogradation (4°C/18 h–30°C/6 h), on the physiology and behavior of mice. HR0 starch exhibited low in vitro and in vivo glycemic indexes of 37 and 51, respectively. Mice fed a high‐fat diet supplemented with 33% HR0 starch showed significant reductions in body weight compared to those on a high‐fat diet. HR0 supplementation normalized fasting plasma glucose levels in mice, preventing the development of diabetes, while all other high‐fat diet groups developed diabetic symptoms. Blood lipid analysis indicated that HR0 reduced triglycerides, LDL cholesterol, and total cholesterol levels while increasing HDL cholesterol. Histological examinations showed that HR0 improved the health of liver, kidney, and adipose tissues, notably reducing the incidence of fatty liver disease. Behavioral tests demonstrated that HR0 starch enhanced locomotor activity and reduced anxiety levels. These results suggest that HR0 starch can effectively improve metabolic health and may serve as a beneficial dietary supplement for individuals at risk of obesity, diabetes, and cardiovascular diseases.

## 1. Introduction

Water yam (*Dioscorea alata*) is a widely cultivated tuber crop in several parts of the world. It is an important nutritional and economic resource due to its ease of propagation, high nutritional value, and extended shelf‐life [1]. The starch content of water yam tubers typically ranges from 17% to 28% on a fresh weight basis, with amylose content varying between 29% and 38% [2]. The physicochemical properties of water yam starch, such as gelatinization temperature (70°C–85°C), swelling power, and water absorption capacity, have been found to be comparable to or higher than those of other tuber starches [3]. These properties make water yam starch suitable for various food and industrial applications.

Moreover, several studies have explored modifications of water yam starch to enhance its functional properties. For instance, acetylation has been shown to improve the stability and clarity of water yam starch pastes [4], while oxidation treatment can increase its resistance to retrogradation [5]. To the best of our knowledge, there were no studies about using retrogradation of gelatinized water yam starch to modify its structure and functionality. Starch retrogradation is the reassociation or recrystallization of gelatinized starch into a more ordered structure. This phenomenon can occur during freeze/thaw cycles and significantly alters starch properties, particularly its digestibility. Studies have shown that retrograded starches contain higher levels of resistant starch (RS), a type of starch that resists digestion in the small intestine and functions similarly to dietary fiber [6]. This resistance is primarily attributed to the formation of crystalline structures, especially in the amylose fraction, which are less accessible to digestive enzymes [7]. The extent of retrogradation and its impact on digestibility vary among starch sources; for instance, high‐amylose starches tend to retrograde more extensively and form more RS than their low‐amylose counterparts [8]. The reduced digestibility of retrograded starches has garnered interest in their potential health benefits, including improved glycemic control and enhanced colonic health [9].

The glycemic index (GI) measures how foods affect blood sugar levels, providing information about carbohydrate availability. Low GI diets have shown potential therapeutic and physiological benefits for various populations [10]. GI values are categorized as high (> 70), medium (55–69), and low (< 55). Studies have found that the retrogradation process can increase the content of RS, thereby reducing the GI [11, 12]. Animal studies have demonstrated that RS can have hypoglycemic effects and improve lipid metabolism and body weight [13, 14].

Temperature cycling during storage can enhance retrogradation and RS formation, as demonstrated in studies on various starch sources [15]. Therefore, in this study, gelatinized yam starch was subjected to two cycles of retrogradation (48 h) to obtain the retrograded starch (HR0). The GI of HR0 starch will be evaluated through in vitro and in vivo experiments. Additionally, HR0 starch will be incorporated into normal diet (ND) and high‐fat (HF) diet to assess its effects on the physiology and physical activities of tested mice.

## 2. Materials and Methods

### 2.1. Preparation of Water Yam Starch

Water yam tubers (2–3 kg/tuber) were washed, peeled, and cut into 1 × 1 cm cubes. From 1.0 kg of peeled tubers, 0.88 kg of flesh remained. The tuber cubes were soaked in 0.2% NaOH solution (1:9 w/v), ground, and sieved (200 mesh). The suspension was stirred thoroughly, allowed to settle, and the supernatant was replaced with fresh NaOH solution every 4 h until being negative to the ninhydrin test. The settled sediment was then homogenized with distilled water (DW) and adjusted to pH 7.0 using a 0.1N HCl solution, and then, the supernatant was removed. DW was then added and homogenized with the sediment (starch) and allowed to settle until the supernatant showed no white precipitate when tested with AgNO_3_ solution. The settled starch was dried at 50°C for 24 h until the moisture content was below 10%. The starch was then ground, sieved (200 mesh), and stored in sealed packaging in a dry, cool place until used for further studies [16]. From 1 kg of initial water yam material, 0.11 kg of water yam starch was obtained after processing. The proximate composition of the fresh yam and the yam starch is determined and presented in Table 1.

**Table 1:** Chemical components of fresh water yam tuber and water yam starch.

Components (g/100 g)	Fresh water yam	Water yam starch
Starch	21.60	82.0
Moisture content	71.70	15.0
Crude protein	3.52	ND
Total fat	0.27	0.321
Total ash	0.71	Not detected
Fiber	1.70	2.04

### 2.2. Preparation of Gelatinized and Retrogradated Water Yam Starches

The native yam starch (12 g in 400 mL water) was gelatinized (95°C, 30 min) and autoclaved (121°C, 15 min) and then dried at 50°C for 24 h to obtain the gelatinized starch (*A*).

To produce retrograded yam starch (HR0), the native starch was first gelatinized (95°C, 30 min) and autoclaved (121°C, 15 min). The cooled starch gel was then retrograded through two consecutive 24 h cycles (18 h at 4°C, 6 h at 30°C) [17]. The retrograded starch (HR0) was dried at 50°C for 24 h to reach less than 10% moisture content.

### 2.3. *In Vitro* Digestion and Estimated GI (eGI)

A solution of 10 U/mL *α*‐amylase in 0.1 M phosphate buffer (pH 6.9) was prepared and stabilized at 37°C. Starch samples (0.5 g in 25 mL buffer) were also stabilized at 37°C.

The enzyme solution (5 mL) was added to the starch suspension to initiate hydrolysis. At various time points (0–480 min), samples were taken, mixed with dinitro salicylic acid (DNS) reagent, and heated to stop the reaction. The degree of hydrolysis (DH) was reducing sugar content that determined by comparing the absorbance at 503 nm to a maltose standard curve (Arp et al., 2020) [18].

The hydrolysis curve was plotted and regressed to the following equation:

(1) C=C∞1−e−kt,

where *C* is the reducing sugar content, *C*_*∞*_ is the maximum value, and *k* is the rate constant [19]. The area under the curve (AUC) was calculated for *A* and HR0 using Origin software, and the eGI of HR0 was determined as its percentage AUC relative to that of *A*.

### 2.4. *In Vivo* Experiments

**Figure 1:**
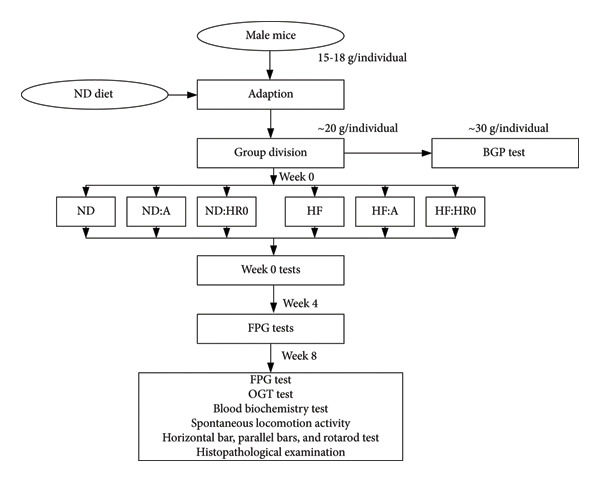
Diagram of in vivo experiments.

The scheme of *in vivo* experiments in this study is presented in Figure 1. Six‐week‐old male albino mice (*Mus musculus*), weighing 15–18 g, were obtained from the Ho Chi Minh City Pasteur Institute, Vietnam. After acclimating to a ND for 1–2 weeks, the mice reached a weight of 20 ± 1 g. They were maintained under standard conditions: 30 ± 1°C temperature, 60%–70% humidity, and a 12‐h light/dark cycle, with food and water provided ad libitum [20]. A vitamin solution (Hagrbuttentrunk, Beaphar, Netherlands) was added to their drinking water daily.

Following acclimatization, the mice were divided into six groups (5 per group) and assigned respective diets for 8 weeks (Table 2). Body weight gain (BWG) and calorie intake (CI) were recorded weekly, starting from Week 0. Fasting plasma glucose (FPG) tests were performed at Week 0, and at the end of Weeks 4 and 8, while the oral glucose tolerance (OGT) test was conducted at the end of Week 8. Blood biochemistry, histopathological analysis, spontaneous locomotion activity, and rotarod tests were carried out at Week 0 and the end of Week 8.

**Table 2:** Components and total calories of the diets.

Compositions (g)	NDs			HFs		
	ND	ND‐A	ND‐HR0	HF	HF‐A	HF:HR0
Fullvit JP70^1^	105.56	52.78	52.78	52.78	24.79	24.79
A	—	35.40	—	—	17.70	—
HR0	—	—	35.40	—	—	17.70
Whey protein^2^	—	7.75	7.75	—	3.88	3.88
Tallow	—	1.63	1.63	43.12	43.93	43.93
Fiber (CMC)	—	8.00	8.00	—	5.60	5.60
Carbohydrate	70.8			35.4		
Crude lipid	3.26			44.75		
Protein	15.5			7.75		
Fiber	16.0			8.0		
Total calories (kcal/100 g)	374.54			575.33		

*Note:* Chemical composition (g/100 g) of whey protein included protein (76.90), crude fat (4.12), starch (3.25), dietary fiber (2.79), total ash (2.73), and moisture (4.23); *A*: gelatinized water yam starch.

^1^Fullvit JP70 (Jolly Pet Products, Ohio, USA).

^2^Impact Whey Protein (Myprotein, USA).

Appropriate sample size is critical in *in vivo* experiments to avoid unreliable results from small groups and waste from excessively large groups [21]. Following the 3R principles and the “resource equation” method [22], a sample size of 5 mice per group was deemed sufficient to ensure reliable outcomes while minimizing animal use.

The *in vivo* experiments were conducted under certificate No. IRB‐A‐2301, approved by the Institutional Review Board at Dinh Tien Hoang Institute of Medicine, which operates under code IRB‐VN0210, and issued by the Vietnam Ministry of Health on October 15, 2015.

In this study, ND and HF diet were used (Table 2). To induce overweight, obesity, and diabetes, the mice were fed HF [23]. Prolonged consumption of HF led to obesity, insulin resistance, and glucose intolerance, increasing the risk of diabetes. The carbohydrate source (50%) in ND was substituted with gelatinized yam starch (*A*) and retrograded yam starch (HR0) to assess their effects on the mice’s physiology, behavior, and locomotion.

### 2.5. Diets for Tested Groups

For diet preparation, tallow was cleaned, cut into small pieces (1 × 1 cm), cooked to liquefy, filtered, and stored at −20°C until use. The mice were divided into two main diet groups: ND and HF. The ND was based on Fullvit JP70 feed (Jolly Pet Products, Ohio, USA), following AIN‐93 diet (Reeves, 1997). The experimental groups received the following diets (Table 2): (1) ND (control); (2) ND‐A (ND with A for 50% of carbs); (3) ND‐HR0 (ND with HR0 as 50% of carbs); (4) HF (HF diet with 70% of energy from fat); (5) HF‐A (HF diet with A for 50% of carbs); (6) HF‐HR0 (HF diet with HR0 as 50% of carbs).

The diets were mixed according to the formula in Table 2. The ND‐A and ND‐HR0 diets were prepared by mixing the dry ingredients (commercial feed, starch, whey protein, CMC) with water, forming bars (1 cm), and drying at 40°C for 24 h. The HF, HF‐A, and HF‐HR0 diets were prepared by melting beef tallow and mixing with the dry ingredients, spreading the mixture thin, and hardening it at 5°C for 3 h. All diets were stored in ziplock bags at 5°C and used within 3 days.

### 2.6. Blood Glucose Profile (BGP) Test

Mice used for the BGP test were housed separately. After being acclimated to a ND until reaching 30 ± 1 g, they were fasted for 12 h (with access to water). Mice were administered 0.5 mL of a 7.5% glucose solution or starch suspensions (A or HR0) via oral gavage (Shin et al., 2007) [24]. Blood samples were collected from the tail vein at 0, 15, 30, 60, 90, 120, and 180 min, and blood glucose levels (BGLs) were measured using a BeneCheck glucose meter (General Life Biotechnology, Taiwan) [25]. The AUC of blood glucose versus time was calculated using both the trapezoid rule (method A) and Origin 8.5.1 software (method B) [26]. The iGI value was estimated by comparing AUC values to glucose (iGI = 100).

### 2.7. FPG and OGT Tests

The FPG and OGT tests, commonly used for diagnosing diabetes and glucose tolerance [27], were performed on fasted mice. For the FPG test, blood from the tail vein was measured using a BeneCheck glucometer [25]. In the OGT test, mice were given 0.5 mL of 7.5% glucose solution, and blood glucose was measured at the same intervals as the BGP test. AUC values were calculated similarly, and the iGI value was determined by comparing the AUC to the ND control (AUC for glucose = 100).

### 2.8. BWG, Total Calorie Intake (TCI), and Food Efficiency Ratio (FER)

BWG (g) and TCI (kcal) were measured weekly. BWG was calculated based on weight gain since week 0, while TCI was based on total food consumption, converted to calories. FER (g/kcal) was calculated by dividing BWG by TCI.

### 2.9. Blood Biochemistry and Histopathology

At week 8, mice were dissected to collect liver, kidney, and fat tissues after cervical dislocation. Approximately 1.0 mL of blood was drawn from the heart and stored in heparin tubes [25]. Blood lipids were analyzed using the AU400 Chemistry Analyzer (Beckman Coulter). Tissues were preserved in 10% formalin, stained with H&E, and examined under a bright‐field microscope.

### 2.10. Spontaneous Locomotion Activity

Mouse movement behavior was analyzed using black acrylic boxes (12 × 12 × 12 inches) and recorded via camera. The videos (10 min, 30 fpm) were analyzed using MATLAB 2019 and a Mouse Activity script [28].

### 2.11. Rotarod Test

The rotarod test was used to assess motor balance and coordination [29]. Mice balanced on a rotating rod (3 cm diameter) accelerating from 4 to 40 rpm over 300 s. Mice underwent three training sessions, followed by up to three test trials per day with 30 min intervals. Time on the rod was recorded, with adjustments made for passive rotations.

### 2.12. Statistical Analysis

All measurements were repeated at least 3 times, and results of calculations were presented as mean ± standard deviation. The analysis of variance methods was conducted with Duncan’s test at a significant level of 0.05 using Minitab Statistical Software (Version 18, Minitab, LLC).

## 3. Results and Discussions

### 3.1. *In Vitro* Digestion and eGI of Starches

**Figure 2:**
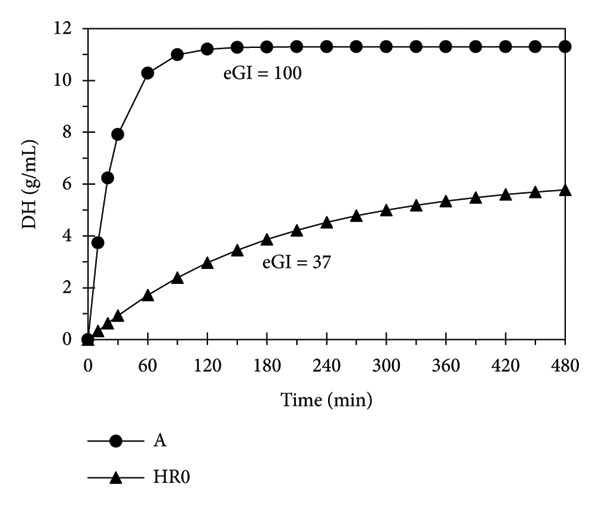
Degree of hydrolysis (DH) and estimated glycemic index (eGI) of starches.

The results of *in vitro* digestion test with *α*‐amylase show that gelatinized starch (*A*) experienced a rapid increase in DH within the first 60 min, reaching a maximum at 120 min, after which it remained stable until 480 min, indicating complete hydrolysis (Figure 2). Therefore, starch A is considered fully hydrolyzed (eGI = 100). In contrast, the HR0 starch exhibited a slower increase in DH and a significantly lower DH after 480 min, with an AUC ratio indicating an eGI of 37, classifying it as a low‐GI starch [30].

Previous studies have shown that retrogradation significantly reduces the GI of starches [31]. In particular, the high amylose content and amylopectin structure in water yam starch promote recrystallization, increasing resistance to alpha amylase hydrolysis [32]. Our own research (data not shown) found that the ratio of alpha helix to amorphous structures in A and HR0 starches was 0.73 and 0.81, respectively. Additionally, the degree of crystallization for A and HR0 starches was 20.35% and 68.07%, respectively, showing that HR0 has more crystalline regions, which hinder enzymatic digestion [33, 34].

### 3.2. BWG and TCI

Table 2 shows that the ND diet provides 374.54 kcal/100 g, while the HF diet provides 575.33 kcal/100 g. As a result, HF diets provide more energy than ND diets when equal food quantities are consumed, and the HF diet’s appealing smell likely increases consumption. According to Figure​ 3, BWG after 8 weeks was highest in HF groups, arranged as follows: ND < ND‐HR0 < ND‐A ≈ HF‐HR0 < HF‐A < HF.

**Figure 3:**
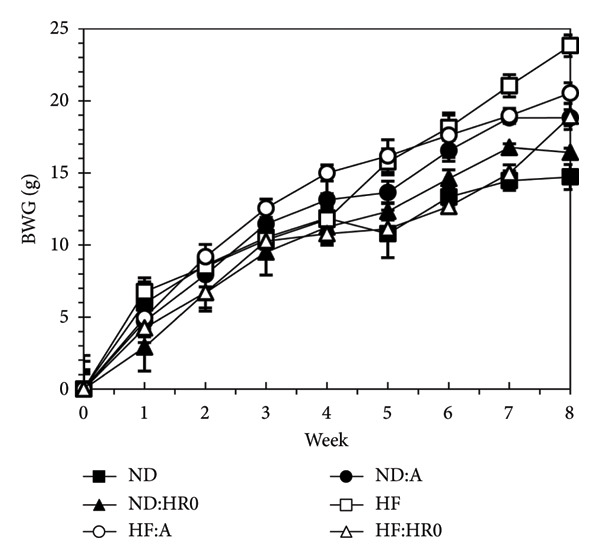
Body weight gain (BWG) of tested groups.

**Figure 4:**
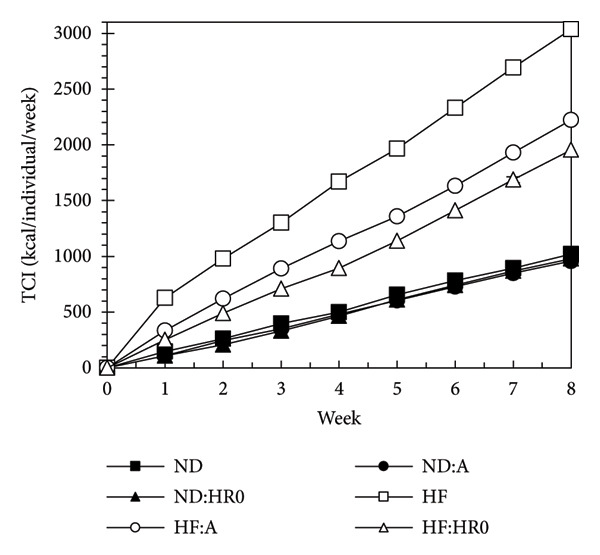
Total calories intake (TCI) of tested groups.

Figure 4 indicates that TCI after 8 weeks followed this order: ND ≈ ND‐A ≈ ND‐HR0 < HF‐HR0 < HF‐A ≈ HF showing that ND groups had lower TCI than HF groups. Supplementing A or HR0 starch reduced TCI significantly in HF groups.

**Figure 5:**
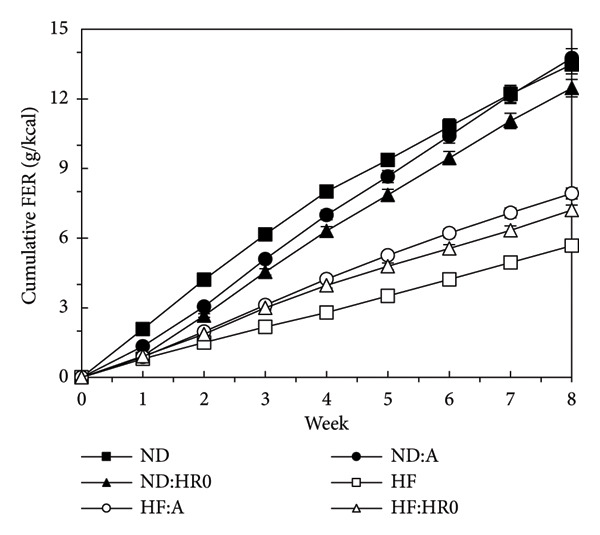
Food efficiency ratio of tested groups.

According to Figure 5, the FER was ordered as follows: HF > HF‐A > HF‐HR0 > ND‐A ≈ ND > ND‐HR0, with HR0 starch more effectively reducing food absorption efficiency compared to A starch [35].

Excess energy from food is stored as adipose tissue, leading to increased body weight [36]. While 1.0 g of starch typically provides 4 kcal, starch types differ in GI, affecting their actual caloric content [37]. HR0 starch, with its lower GI, provides less energy compared to ND‐HR0 and ND‐A starches, explaining the lower BWG in the HR0 group.

### 3.3. BGP Test and *In Vivo* GI (iGI) of Starches

**Figure 6:**
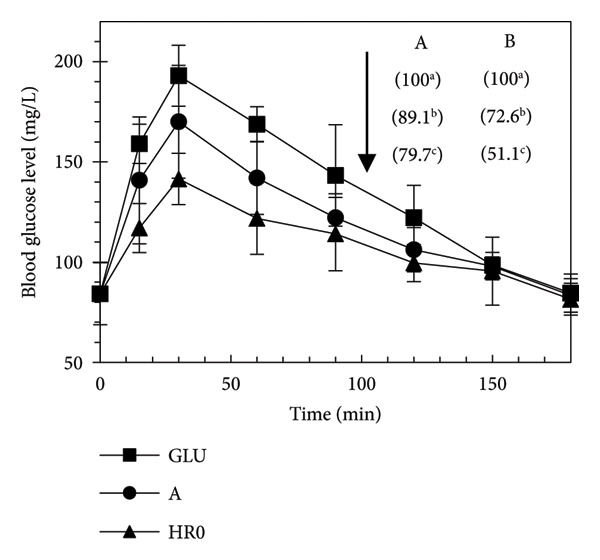
Blood glucose profile (BGP) of tested groups after feeding of glucose or starches (Number in brackets was the iGI of each sample calculated in either methods A or B; GLU was glucose).

Figure 6 presents the results of the BGP test, where mice were fed glucose, gelatinized A starch, or HR0 starch. Initial BGLs at 0 min (∼84.25 mg/L) showed no significant differences among the groups (*p* < 0.05). All groups experienced a rapid rise in BGL, peaking at 30 min. The peak BGL (BGLmax) was ranked as follows (*p* < 0.05): glucose (193 mg/L) > A starch (170 mg/L) > HR0 starch (141.5 mg/L). BGLs gradually decreased from 30 to 180 min, returning to baseline levels, with HR0 starch consistently showing the lowest BGL throughout the test.

The GI values for the groups followed this order (Figure 6): glucose > A starch > HR0 starch. The HR0 starch had an iGI of 51.1 (< 55, based on method B), indicating a significantly lower glycemic response compared to A starch. Differences between the eGI and in vivo glycemic index (iGI) arise from different conditions in vitro versus in vivo. The eGI is based on the hydrolysis of starch by alpha amylase, producing dextrin, whereas the iGI reflects glucose levels in the bloodstream, following starch digestion by enzymes in the animal’s digestive system.

iGI measurements are influenced by additional factors such as the food’s physical form, nutrient content, and physiological factors like digestibility and glucose absorption. These are not accounted for in vitro eGI testing, which focuses solely on hydrolysis reactions. Gastric emptying rate, a major determinant of digestion speed in the small intestine, also plays a critical role in vivo, affecting the results [38].

The HR0 starch used in this study shows promise in controlling diabetes and reducing cardiovascular risk [39]. Previous research has shown that low GI foods (< 55) help regulate blood glucose and improve lipid profiles compared to high GI foods (> 70) [40]. In conclusion, determining the GI involves both predictive in vitro testing and confirmatory in vivo testing.

### 3.4. FPG Test

**Figure 7:**
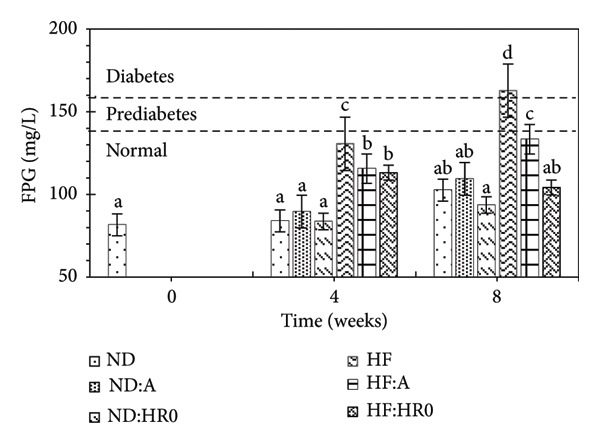
Fasting plasma glucose (FPG) level of tested groups.

The FPG test is used to assess glucose absorption and diagnose diabetes [41]. The FPG values of the experimental groups are shown in Figure 7. Over the course of 4 and 8 weeks, all groups exhibited an increase in FPG compared to baseline. The ND, ND‐A, and ND‐HR0 groups remained within the normal FPG range (< 130 mg/L), while the HF group exceeded this threshold (130.60 mg/L) after 4 weeks, indicating early prediabetes [42]. All HF groups had significantly higher FPG than the ND groups.

After 8 weeks, FPG values for the ND groups were < 130 mg/L, indicating normal glucose levels. In contrast, the HF group’s FPG rose to 162.80 m/L, signifying type 2 diabetes (T2D). The HF‐A group displayed prediabetic levels, while HF‐HR0 remained in the normal range. The FPG hierarchy among HF diet groups was as follows: HF (162.80 mg/L) > HF‐A (133.40 mg/L) > HF‐HR0 (104.20 mg/L), demonstrating the effectiveness of HR0 starch in mitigating T2D risk.

### 3.5. OGT Test

**Figure 8:**
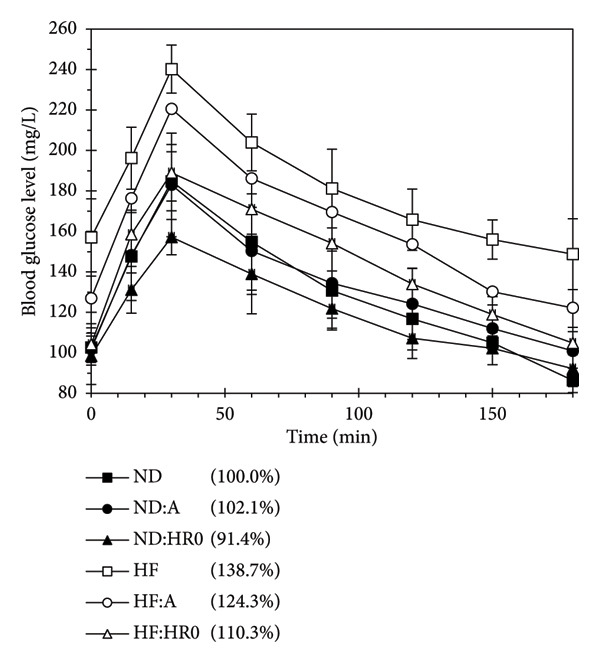
Oral glucose tolerance test of tested groups. (in the brackets: ratio of AUC of tested group compared to ND).

OGT test results (Figure 8) revealed that all groups reached peak blood glucose (BGLmax) 30 min after consuming glucose, followed by a return to baseline within 180 min. The BGL and BGLmax values (*p* < 0.01) were ranked as ND‐HR0 < ND‐A ≈ ND ≈ HF‐HR0 < HF‐A < HF, with HF groups consistently showing higher BGL than ND groups.

The AUC for BGLs (Figure 8) represents the total glucose content over 180 min [41]. AUC rankings were as follows: ND‐HR0 < ND < ND‐A < HF‐HR0 < HF‐A < HF, with values expressed as a percentage relative to ND. Group ND‐A had a higher AUC than ND, while ND‐HR0 had a significantly lower AUC than both ND and ND‐A, indicating HR0’s lower energy contribution compared to other starches. Conversely, HF had a significantly higher AUC than ND, indicating impaired blood sugar control. Starches A and HR0 both reduced blood glucose in HF groups, showing that HR0, and to some extent A, improved glycemic control in HF diet mice, though starch A had varied effects depending on the diet. HR0 was particularly effective in managing blood sugar in HF diet mice.

A previous study found that increased FPG in obese individuals may indicate a higher risk of developing nonalcoholic fatty liver disease (NAFLD) [43]. Long‐term consumption of a HF diet impairs insulin receptor function, leading to an increased risk of T2D. This diet promotes weight gain, which contributes to insulin resistance, a condition where cells become less responsive to insulin, raising BGLs and eventually leading to T2D [44, 45]. Obesity, a major T2D risk factor, plays a central role in its development [46, 47]. However, the relationship between dietary fat, diabetes, and insulin is complex and influenced by genetic and nutritional factors [44, 45].

Low GI foods have been shown to help control T2D by slowing carbohydrate absorption, reducing the risk of diabetes, hypertension, and cardiovascular disease. Replacing high GI foods with low GI alternatives offers clinically significant benefits in blood sugar control, comparable to those of medications for postprandial glucose regulation [38, 48, 49].

### 3.6. Blood Lipid Parameters

The insulin values of all tested groups when fasting were < 4 UI/mL (data not shown). Meanwhile, the FPG levels of the tested groups (Figure 7) showed differences. Therefore, in this study, the insulin index did not accurately reflect the diabetic condition of the experimental animals.

Total cholesterol (TC), consisting of LDL‐C and HDL‐C, ranged from 1.743–4.665 mmol/L, within normal limits for mice [50, 51]. At 8 weeks, TC levels were ranked as follows (*p* < 0.05): ND‐HR0 ≈ ND < ND‐A < HF‐A ≈ HF‐HR0 < HF, showing that a HF diet raised TC, while HR0 starch significantly reduced it. Additionally, LDL‐C and HDL‐C levels were higher in HF groups than in ND groups. Notably, HR0 and A starches in HF diets reduced LDL‐C and increased HDL‐C, respectively.

Both A and HR0 starches significantly reduced triglyceride (TG) levels compared to HF (Table 3). TG, which accounts for 95% of daily fat intake, is stored in adipose and liver tissues, with normal levels for mice between 0.8 and 1.85 mmol/L [50, 51]. All groups fell within this range. High‐fat diets promote TG accumulation in the liver, leading to hepatic steatosis, insulin resistance, and dyslipidemia [52]. This study also suggests that changes in TC are linked to increased insulin resistance and dyslipidemia, while HDL‐C levels are associated with higher fat intake, particularly monounsaturated fats.

**Table 3:** Blood lipid parameters.

Group	TG (mmol/L)	HDL‐C (mmol/L)	LDL‐C (mmol/L)	TC (mmol/L)
ND	1.07 ± 0.11^bc^	1.40 ± 0.13^c^	0.42 ± 0.08^c^	1.94 ± 1.00^d^
ND‐A	1.00 ± 0.13^c^	2.17 ± 0.11^b^	0.43 ± 0.04^c^	2.73 ± 0.18^c^
ND‐HR0	1.21 ± 0.12^b^	1.10 ± 0.11^d^	0.54 ± 0.04^c^	1.77 ± 0.08^d^
HF	1.69 ± 0.06^a^	2.83 ± 0.09^a^	1.28 ± 0.11^a^	4.43 ± 0.09^a^
HF‐A	0.57 ± 0.08^d^	2.79 ± 0.13^a^	0.80 ± 0.10^b^	3.80 ± 0.12^b^
HF:HR0	0.56 ± 0.05^d^	2.64 ± 0.08^a^	0.83 ± 0.04^b^	3.68 ± 0.11^b^

*Note:* Values (mean) with different superscript characters in the same column indicate a statistically significant difference (*p* < 0.05).

Previous research demonstrated that RS (low GI) from corn/rice lowers cholesterol in diabetic mice [13]. This starch ferments in the colon, producing short‐chain fatty acids (SCFAs), which inhibit cholesterol synthesis in the liver and intestines [53].

### 3.7. Tissue Weight and Histopathological Studies of Liver, Kidney, White Adipose, Gastrocnemius, and Soleus Tissues

Previous studies have shown that a HF diet can lead to increased abdominal fat, which is linked to insulin resistance, dyslipidemia, hypertension, T2D, cardiovascular disease, colorectal cancer, and cognitive decline [47, 52, 54]. Visceral fat accumulation, surrounding the abdominal organs, is also a consequence of HF diets [55].

According to Table 4, the white adipose tissue weights after 8 weeks were ranked as follows (*p* < 0.05): ND ≈ ND‐A ≈ ND‐HR0 < HF‐HR0 ≈ HF‐A < HF. The FW/BW ratio followed a similar trend (*p* < 0.05): ND ≈ ND‐HR0 ≈ ND‐A < HF‐HR0 < HF ≈ HF‐A. These significant differences between HF and ND groups indicate that a HF diet promotes white adipose tissue accumulation, leading to overweight and obesity. HR0 starch reduced white adipose tissue accumulation, as low GI starch enhances fat oxidation and reduces fat storage [56]. RS diets have also been associated with reduced fat mass (Nugent, 2005), and low GI diets may lower body fat and improve metabolic response in T2D [57, 58].

**Table 4:** Weight of tissues.

Groups	FW (g)	LW (g)	KW (g)	FW/BW (%)	LW/BW (%)	KW/BW (%)
ND	1.10 ± 0.49^c^	1.32 ± 0.08^c^	0.39 ± 0.04^ab^	3.30 ± 1.44^c^	4.01 ± 0.23^a^	1.19 ± 0.13^a^
ND‐A	1.15 ± 0.21^c^	1.48 ± 0.17^bc^	0.36 ± 0.07^abc^	4.30 ± 1.23^c^	4.39 ± 0.44^a^	1.24 ± 0.06^a^
ND‐HR0	1.19 ± 0.06^c^	1.28 ± 0.14^c^	0.30 ± 0.02^bcd^	3.49 ± 0.14^c^	3.74 ± 0.44^a^	0.89 ± 0.05^b^
HF	8.35 ± 1.11^a^	1.88 ± 0.04^a^	0.43 ± 0.07^a^	19.81 ± 2.84^a^	4.43 ± 0.22^a^	1.06 ± 0.14^ab^
HF‐A	7.90 ± 0.43^b^	1.82 ± 0.08^ab^	0.24 ± 0.02^cd^	19.89 ± 0.70^a^	4.61 ± 0.38^a^	0.60 ± 0.05^c^
HF:HR0	5.16 ± 1.78^b^	1.55 ± 0.13^abc^	0.21 ± 0.02^d^	13.89 ± 4.52^b^	4.28 ± 0.49^a^	0.59 ± 0.09^c^

*Note:* Values with different symbols in the same column indicate a statistically significant difference (*p* < 0.05); FW, fat (white adipose) tissue weight.

Abbreviations: KW, kidney weight; LW, liver weight.

Liver weights (LW) and LW/BW ratios did not differ significantly between groups. Similarly, kidney weights (KWs) did not vary between ND and HF groups, though KW values for HF‐A and HF‐HR0 were lower. KW/BW ratios for ND‐HR0 and HF‐HR0 were significantly lower than in their respective control groups, suggesting that HR0 starch reduced KW.

**Figure 9:**
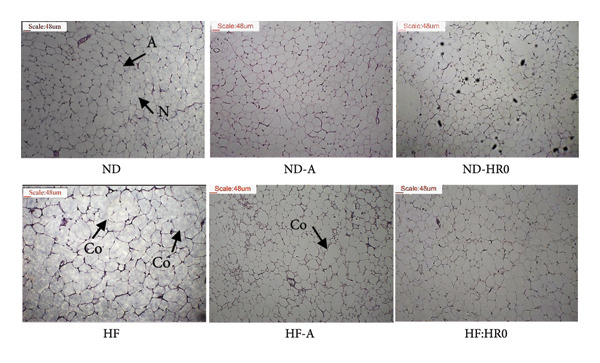
Photomicrographs of white adipose tissue (H&E stained sections; ×100; A: Adipocytes; Co: Coalescence; N: Nucleus).

Histopathological analysis (Figure 9) showed that adipose cell sizes in the ND and ND‐A groups were normal, with no signs of coalescence. In contrast, the HF group exhibited larger adipose cells with overlapping and coalescence, indicative of obesity. HF‐A and HF‐HR0 groups showed smaller adipose cells than the HF group, demonstrating that HR0 starch significantly reduced white adipose cell size in HF diet mice.

**Figure 10:**
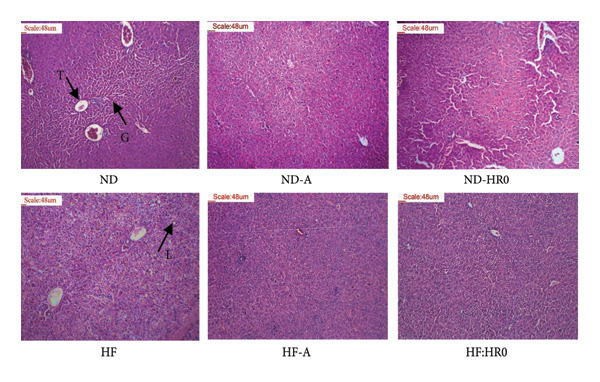
Photomicrographs of liver tissue (H&E stained sections; ×100; T: Central vein; G: Hepatocytes; L: Lipid droplet).

Liver histology (Figure 10) revealed normal liver cell morphology in ND groups, while HF groups showed significant fat droplet accumulation (L), a hallmark of NAFLD [47]. However, supplementing HF diets with A starch and HR0 starch reduced or eliminated these fat deposits, indicating that low GI starch promotes fat metabolism in the liver [59]. HR0 starch also reduced LW and fat accumulation in HF diet groups.

**Figure 11:**
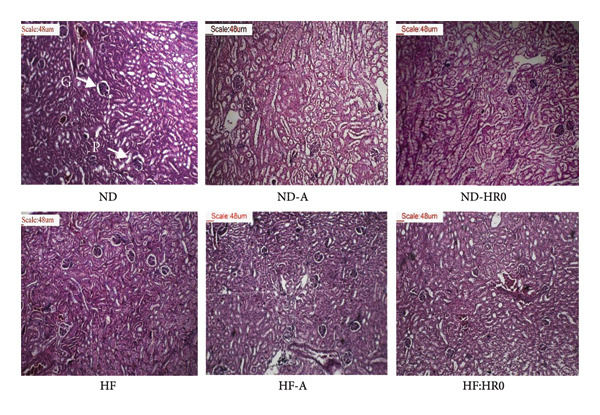
Photomicrographs of kidney tissue (H&E stained sections, ×100; G: Glomerulus; P: Proximal convoluted tubule).

Kidney tissue analysis (Figure 11) showed no abnormalities in any of the tested groups.

### 3.8. Spontaneous Locomotion Activity

**Figure 12:**
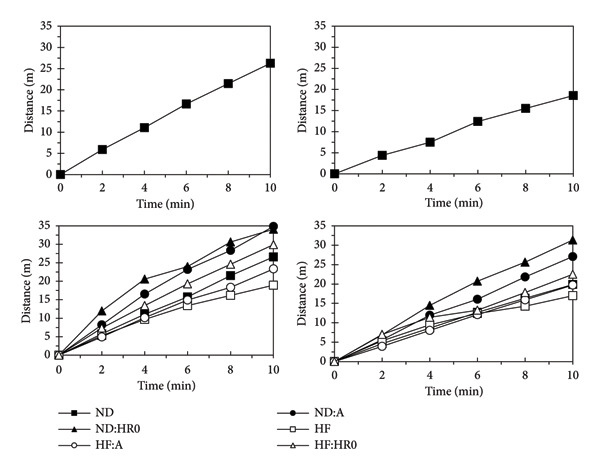
The travel distance of tested mice before (left) and after (right) feeding at 0 week (the 2 charts above) and 8 weeks (the 2 charts below).

At week 0, the distance traveled by mice after feeding (AF) was 70.7% of the distance traveled before feeding (BF) (Figure 12). The mice were then divided into six groups with different diets. After 8 weeks, the BF traveled distance was ranked as follows (*p* < 0.05): HF < HF‐A < ND < HF‐HR0 < ND‐A ≈ ND‐HR0. Similarly, the AF traveled distance ranked as HF < HF‐A ≈ ND < HF‐HR0 < ND‐A < ND‐HR0. In all cases, the AF distance was shorter than the BF distance, with ND‐HR0 showing the highest HF and the lowest traveled distance. Starch and HR0 supplementation increased mobility and exploratory behavior, especially in the HF diet groups.

A study by Radulian et al. highlighted that low GI diets lead to favorable metabolic effects, such as weight loss, improved fasting glucose, reduced TGs, and better blood pressure, potentially influencing locomotion [58]. Heung‐Sang Wong et al. demonstrated that low GI diets improve exercise performance, likely due to more stable glucose and free fatty acid levels during activity [60].

**Figure 13:**
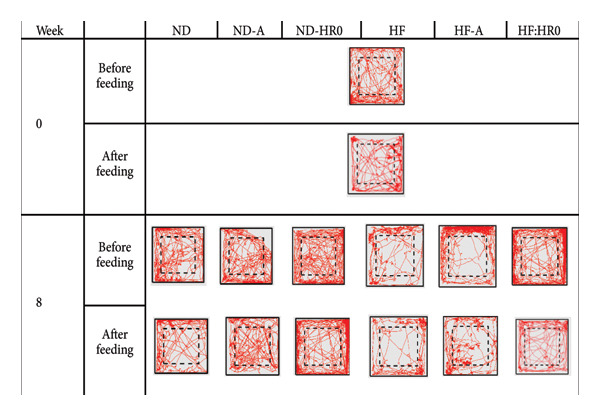
The movement trajectory of the tested mice.

Figure 13 shows the movement trajectories at the start and end of the study. In all groups, BF movement and center zone activity were greater than AF. The ND groups exhibited more movement and center zone activity than the HF groups. Among the HF groups, diets with A and HR0 starch increased both overall and center zone movement compared to the HF group.

These results confirm that mice move less AF. In both the ND‐HR0 and HF‐HR0 groups, the mice also showed more wall‐hugging behavior (thigmotaxis), possibly due to HR0 starch’s low GI, which may cause mild digestive discomfort, affecting their behavior.

A previous study suggested that nervous system changes can reduce exploratory behavior, increasing stress and anxiety, leading to reduced mobility [61]. Mice that spend more time along the walls and less time in the center are considered more [62]. Reduced center zone activity, often associated with obesity and diabetes, may reflect anxiety‐like behaviors in these metabolic conditions [47, 63].

### 3.9. Rotarod Test

**Figure 14:**
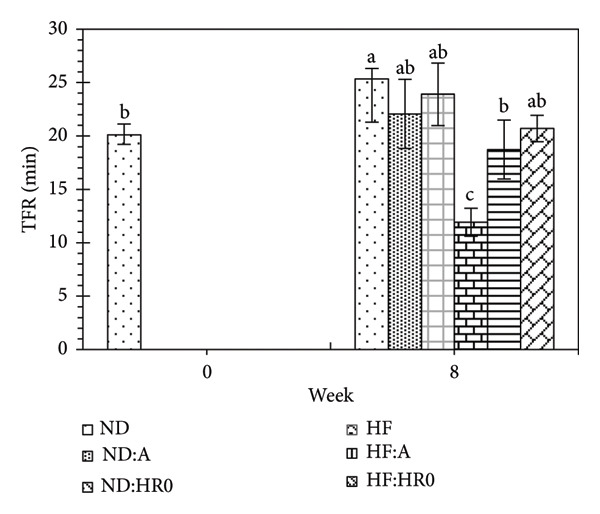
Time until fall of mice on the rotarod (TFR).

The time spent on the rotarod, which measures coordination and muscular endurance, showed significant differences among groups (Figure 14). The ranking of time to fall on the rotarod (TFR) was as follows (*p* < 0.05): HF < HF‐A < HF‐HR0 ≈ ND‐A < ND ≈ ND‐HR0. In the HF diet groups, the HF‐HR0 group had the longest running time, 1.22 times longer than HF and 1.16 times longer than HF‐A. Similarly, in the ND groups, ND‐HR0 had the longest exercise duration. A study by Scribner et al. found that a low GI starch diet enhances fat utilization during sustained physical activity, improving performance and endurance [64].

This study possesses high internal validity, achieved by the use of a genetically homogenous mouse model (Albino mice) and strict control over environmental conditions and dietary components. This ensures that the observed differences between groups can be primarily attributed to the intervention with different types of starch, particularly HR0. However, we acknowledge several limitations that should be considered when interpreting the results. A primary limitation is the inherent physiological and metabolic differences between mice and humans, which warrants caution in directly extrapolating these findings to the human population. The 8‐week study duration, while sufficient for observing acute and mid‐term metabolic changes in mice, may not fully reflect the long‐term effects of HR0 consumption on chronic diseases in humans. Furthermore, the observed thigmotaxis in the HR0‐fed groups, hypothesized to be due to mild digestive discomfort, was not quantitatively assessed using direct methods such as gut microbiota or fermentation product analysis.

Despite these limitations, our study provides strong, first‐time preclinical evidence of the potential of retrograded water yam starch (HR0) to improve metabolic health. These findings are consistent with the broader body of evidence showing that low GI diets are beneficial for managing diabetes and reducing cardiovascular risk [30, 58]. Therefore, this research establishes a solid scientific foundation for subsequent steps. Future directions should include (1) longer‐term animal studies to assess sustained efficacy and safety; (2) mechanistic studies focusing on the effects of HR0 on the gut microbiome and fermentation; (3) direct comparative studies evaluating the effectiveness of HR0 against other commercial RSs or low GI starches; and, most importantly, (4) progressing to controlled human clinical trials to confirm these benefits in individuals at risk of, or with, metabolic disorders.

## 4. Conclusion

Retrograded HR0 starch, processed using cyclic heat retrogradation (4°C/18 h–30°C/6 h) for two cycles, has a low GI. The *in vivo* experiments on mice demonstrated that HR0 starch not only regulates blood sugar but also controls weight and prevents obesity. High‐fat diet mice supplemented with HR0 starch showed (i) significantly improved BGLs; (ii) reduced TG, TC, LDL‐C, and increased HDL‐C, reducing cardiovascular risk; (iii) improved tissue structure affected by obesity; and (iv) increased mobility and reduced anxiety. HR0 starch, with its low GI, has potential for inclusion in foods like cookies, crackers, bread, and snacks for individuals on diets or those managing obesity and diabetes.

## Conflicts of Interest

The authors declare no conflicts of interest.

## Funding

This study was financially supported by Ministry of Education and Training of Vietnam under grant B2024‐SPK‐03 and hosted by Ho Chi Minh City University of Technology and Education, Vietnam.

## Data Availability

The data that support the findings of this study are available from the corresponding author upon reasonable request.
